# Telemedicine via Continuous Remote Care: A Proactive, Patient-Centered Approach to Improve Clinical Outcomes

**DOI:** 10.2196/23646

**Published:** 2021-11-02

**Authors:** Sarah Hallberg, David Harrison

**Affiliations:** 1 Virta Health West Lafayette, IN United States; 2 Virta Health Boston, MA United States; 3 Department of Medicine and Pediatrics Massachusetts General Hospital Boston, MA United States

**Keywords:** telemedicine, continuous remote care, diabetes, COVID-19, pandemic

## Abstract

The COVID-19 pandemic has revolutionized health care for patients and providers alike. Telemedicine has moved from the periphery of our health care system to center stage more rapidly than anyone could have envisioned. Currently, virtual care has quite effectively replicated the traditional health system’s care delivery model and reimbursement structure—a patient makes an appointment, then sees a physician (except with video or phone replacing in-office visits) who makes a care plan, and the patient and physician meet again at a later timepoint to assess progress. Replicating this episodic care paradigm virtually has been invaluable for delivering care swiftly during the COVID-19 pandemic; however, we can and should do more with the connectedness and convenience that telemedicine technology enables. Continuous remote care, with a data-driven, proactive outreach to patients, represents a decisive step forward in contrast to the currently available episodic, reactive, patient-initiated care. In the context of continuous remote care, patient biometric and symptom data (patient entered and connected data) are assimilated in real time by artificial intelligence–enabled clinical platforms to bring physicians' and other health care team members’ attention to those patients who need intervention, whether this is via medication adjustments, acute care management, or lifestyle coaching. In this paper, we discuss how an innovative continuous remote care approach has improved outcomes in another deadly pandemic—type 2 diabetes mellitus.

## Background

The COVID-19 pandemic has revolutionized health care for patients and providers alike. Telemedicine has moved from the periphery of our health care system to center stage more rapidly than anyone could have envisioned. Physicians and health care organizations alike have stepped up to the challenge of swiftly adapting their practices and organizations to virtual care. In the current setting, virtual care has quite effectively replicated the traditional health system’s care delivery model and reimbursement structure, in that a patient first makes a medical appointment, then sees a physician (except with video or phone replacing in-office visits) who makes a care plan, and the patient and physician meet again weeks or months later to assess progress. 

Replicating this episodic care paradigm virtually has been invaluable for delivering care swiftly during the COVID-19 pandemic; however, we can and should do more with the connectedness and convenience that telemedicine technology enables. Despite many advances in medicine, including the ever-growing options for connectivity, we have not realized the improvement in clinical outcomes as chronic diseases continue to be an unabated public health concern.

## Continuous Remote Care

### Overview

An option for a more patient-centered telemedicine experience is continuous remote care (CRC). This approach includes a data-driven, proactive outreach to patients, which represents a decisive step forward in contrast to the currently available episodic, reactive, patient-initiated care. In CRC, patient biometric and symptom data (ie, patient entered and connected data) are assimilated in real time by artificial intelligence–enabled clinical platforms to bring physicians’ and other health care team members’ attention to those patients who need intervention, whether this is via medication adjustments, acute care management, or lifestyle coaching. The care team can review hundreds of patients a day instead of just one or two dozen; proactively engage the patient to develop a care plan; and support patient empowerment, safety, and achievement of optimal health outcomes. As a result of the CRC model’s connectedness, the care team can reassess progress in real time.

In this paper, we discuss how this innovative CRC approach has improved outcomes in another deadly pandemic—type 2 diabetes mellitus.

### CRC for Type 2 Diabetes Mellitus

Patients with chronic diseases such as diabetes require ongoing, aggressive, team-based management to optimize health outcomes and ongoing support to ensure long-term adherence to their care plan. Patients with diabetes must make decisions many times a day related to their care, and for those on insulin and other diabetes medications, each decision can be quite complex. Furthermore, these decisions impact what will happen to the trajectory of their disease over time and may determine if they are destined to have a diabetes-related emergency and need to use emergency services at that time. This type of complex disease is an ideal fit for the CRC approach, as it requires more than the episodic care that our health care system has built upon in order to address the day-to-day, meal-to-meal needs of patients.

Advances in diabetes technology, such as blood sugar monitoring apps and continuous glucose meters, have already improved patient care [1,2]. However, provider advice based on data from these devices is generally delivered during traditional episodic visits. Asynchronous virtual feedback from a health coach or certified diabetes educator has been shown to add additional improvements in glucose control [3,4]. To date, the most significant improvements in glucose control have been seen when remote monitoring is tied to a physician-led care team, as our published results demonstrate [5].

**Figure 1:**
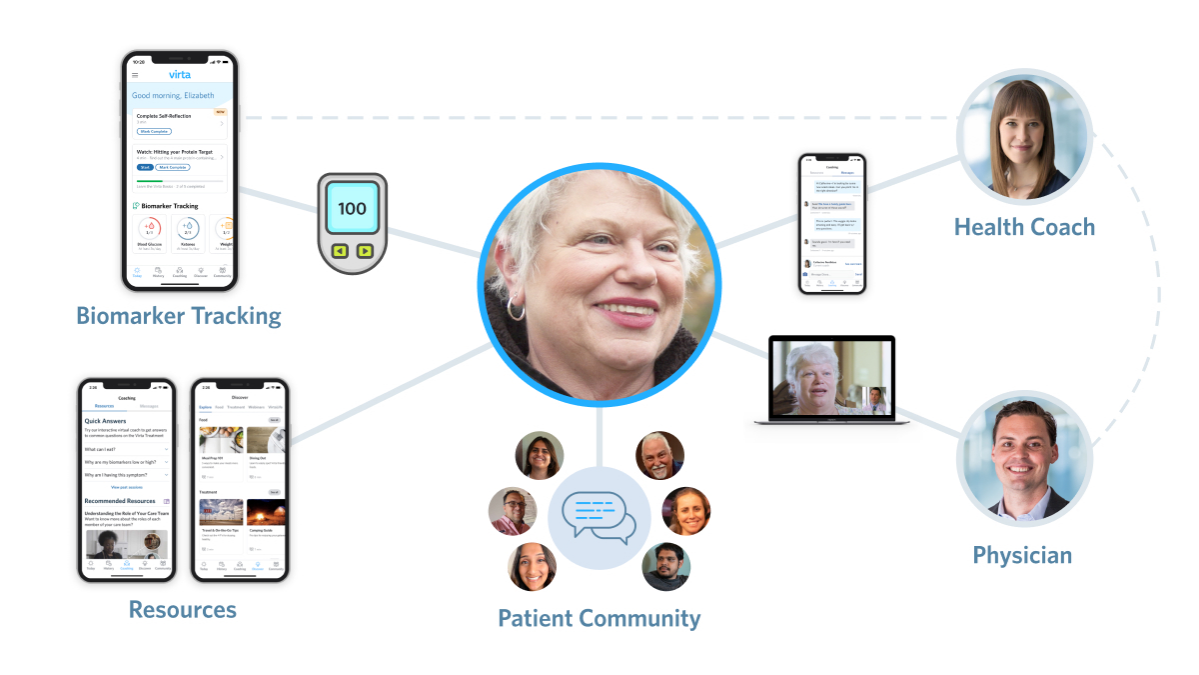
Multifaceted holistic care offered by the continuous remote care model.

Our CRC model was created to provide multifaceted, holistic patient support, ranging from medication adjustments to transformational lifestyle changes (Figure 1). Physicians are alerted by algorithmic prioritization to situations that may be a safety concern or where medications need to be adjusted based on incoming biomarkers, thereby allowing proactive outreach to patients. Health coaches operate in pods so that many specialists (eg, nurse practitioners, behavioral health providers, dieticians, and exercise physiologists) allow for specialized and personalized care for each individual by using the same prioritization system as physicians to guide lifestyle interventions. This high-touch care, including personalized nutrition advice, has allowed for medication de-escalation, often culminating in complete elimination of medication [5]. Moreover, integral to CRC are moderated peer-support groups and readily accessible patient resources that include education on many topics, such as recipe and menu planning, and behavior-focused content. This model has shown a retention and engagement rate of 74% at a 2-year timepoint [5].

### CRC for Other Chronic Diseases

Proactive outreach to patients based on incoming biomarker data has successfully been used for congestive heart failure (CHF) in many trials, with results demonstrating decreased hospitalization rates for CHF exacerbations and lower mortality [6,7]. This model can also track biomarkers for other chronic diseases, such as obesity and hypertension.

## Barriers to Widespread Use

As CRC for diabetes and other chronic diseases continues to evolve, with increasing evidence for its efficacy increases and new indications for this care paradigm being established, the associated reimbursement models will need to evolve as well. Although CRC includes elements of evaluation and management episodes, as well as remote physiologic monitoring, its high frequency of asynchronous patient-provider interactions and the complexity of ongoing behavioral support are not readily captured under or even fully recognized by existing Current Procedural Terminology (CPT) codes. Therefore, development of CRC-specific CPT codes will incentivize adoption of this resource-intensive, but remarkably effective, care paradigm. A CRC paradigm also aligns with the essence and implementation of risk-based reimbursement, as utilized, for example, in Medicaid managed care plans. This would efficiently empower patients to achieve and maintain better health, thereby resulting in fewer costly episodic health services.

Although 90% of Americans now have access to the internet [8], the digital divide remains significant. Those without internet access are particularly vulnerable and are among the populations with the highest rate of diabetes incidence. Working toward universal connectivity and developing CRC programs that can be accessible even by patients with low technology literacy is essential if CRC is to promote real health equity.

## Conclusions

A CRC paradigm, beyond merely providing increased convenience and lower cost of episodic telemedicine care, brings the opportunity for improved patient care and outcomes. A proactive, data-driven, team approach has already been demonstrated to be sustainably adopted by patients and to dramatically improve diabetes and CHF outcomes. This model may also improve outcomes across a wide range of chronic diseases, such as obesity and hypertension, among others, where treatment decisions should be made on incoming data quickly to engage patients and obtain the best health improvements. As we embrace this innovative care model and overcome barriers to its universal use, we can create a new era in which patients are not just the recipients of health care but also the agents and champions of good health.

## Abbreviations

CHF: congestive heart failure
CPT: Current Procedural Terminology
CRC: continuous remote care

## References

[1] Cui M, Wu X, Mao J, Wang X, Nie Min (2016). T2DM self-management via smartphone applications: a systematic review and meta-analysis. PLoS One.

[2] Maiorino MI, Signoriello S, Maio A, Chiodini P, Bellastella Giuseppe, Scappaticcio Lorenzo, Longo Miriam, Giugliano Dario, Esposito Katherine (2020). Effects of continuous glucose monitoring on metrics of glycemic control in diabetes: a systematic review with meta-analysis of randomized controlled trials. Diabetes Care.

[3] Bergenstal RM, Layne JE, Zisser H, Gabbay RA, Barleen NA, Lee AA, Majithia AR, Parkin CG, Dixon RF (2021). Remote application and use of real-time continuous glucose monitoring by adults with type 2 diabetes in a virtual diabetes clinic. Diabetes Technol Ther.

[4] Bollyky JB, Melton ST, Xu T, Painter SL, Knox B (2019). The effect of a cellular-enabled glucose meter on glucose control for patients with diabetes: prospective pre-post study. JMIR Diabetes.

[5] Athinarayanan SJ, Adams RN, Hallberg SJ, McKenzie AL, Bhanpuri NH, Campbell WW, Volek JS, Phinney SD, McCarter JP (2019). Long-term effects of a novel continuous remote care intervention including nutritional ketosis for the management of type 2 diabetes: a 2-year non-randomized clinical trial. Front Endocrinol (Lausanne).

[6] Nakamura N, Koga T, Iseki H (2013). A meta-analysis of remote patient monitoring for chronic heart failure patients. J Telemed Telecare.

[7] Koehler F, Koehler K, Deckwart O, Prescher S, Wegscheider K, Kirwan B, Winkler S, Vettorazzi E, Bruch L, Oeff M, Zugck C, Doerr G, Naegele H, Störk S, Butter C, Sechtem U, Angermann C, Gola G, Prondzinsky R, Edelmann F, Spethmann S, Schellong Sm, Schulze Pc, Bauersachs J, Wellge B, Schoebel C, Tajsic M, Dreger H, Anker Sd, Stangl K (2018). Efficacy of telemedical interventional management in patients with heart failure (TIM-HF2): a randomised, controlled, parallel-group, unmasked trial. The Lancet.

[8] Internet/broadband fact sheet. Pew Research Center.

